# Malaria diagnostic testing and treatment practices in three different *Plasmodium falciparum *transmission settings in Tanzania: before and after a government policy change

**DOI:** 10.1186/1475-2875-10-76

**Published:** 2011-04-02

**Authors:** Guido JH Bastiaens, Erik Schaftenaar, Arnold Ndaro, Monique Keuter, Teun Bousema, Seif A Shekalaghe

**Affiliations:** 1 Nijmegen Institute for International Health, Radboud University Nijmegen Medical Centre, Nijmegen, the Netherlands; 2 Department of Community Health, Kilimanjaro Christian Medical Centre, Moshi, Tanzania; 3 Department Immunology & Infection; Faculty of Infectious & Tropical Diseases, London School of Hygiene & Tropical Medicine, London, UK; 4 Kilimanjaro Christian Medical College, Kilimanjaro, Tanzania; 5 Ifakara Health Institute, Bagamoyo Research and Training Centre, Tanzania

## Abstract

**Background:**

Patterns of decreasing malaria transmission intensity make presumptive treatment of malaria an unjustifiable approach in many African settings. The controlled use of anti-malarials after laboratory confirmed diagnosis is preferable in low endemic areas. Diagnosis may be facilitated by malaria rapid diagnostic tests (RDTs). In this study, the impact of a government policy change, comprising the provision of RDTs and advice to restrict anti-malarial treatment to RDT-positive individuals, was assessed by describing diagnostic behaviour and treatment decision-making in febrile outpatients <10 years of age in three hospitals in the Kagera and Mwanza Region in northern Tanzania.

**Methods:**

Prospective data from Biharamulo and Rubya Designated District Hospital (DDH) were collected before and after policy change, in Sumve DDH no new policy was implemented. Diagnosis of malaria was confirmed by RDT; transmission intensity was evaluated by a serological marker of malaria exposure in hospital attendees.

**Results:**

Prior to policy change, there was no evident association between the actual level of transmission intensity and drug-prescribing behaviour. After policy change, there was a substantial decrease in anti-malarial prescription and an increase in prescription of antibiotics. The proportion of parasite-negative individuals who received anti-malarials decreased from 89.1% (244/274) to 38.7% (46/119) in Biharamulo and from 76.9% (190/247) to 10.0% (48/479) in Rubya after policy change.

**Conclusion:**

This study shows that an official policy change, where RDTs were provided and healthcare providers were advised to adhere to RDT results in prescribing drugs can be followed by more rational drug-prescribing behaviour. The current findings are promising for improving treatment policy in Tanzanian hospitals.

## Background

Malaria accounts for more than 800,000 deaths a year worldwide [1], of which 90% are in young African children [2]. It is the leading cause of health service attendance in Tanzania and comprises ~40% of all morbidity in Tanzanian outpatients below five years of age [3]. This poses a considerable burden on health systems.

Since the 1980s, malaria control has incorporated presumptive treatment of fever with anti-malarials, treating all febrile episodes suspected of malaria with a full therapeutic dose of anti-malarials, chloroquine (CQ, until 2001 in Tanzania [4]) or sulphadoxine-pyrimethamine (SP, until 2007 [5]). This strategy was implemented to effectively treat true cases of malaria and may also have had beneficial effects on malaria transmission by effectively providing chemoprophylaxis to children in malaria endemic areas [6]. However, presumptive treatment may no longer be a justifiable approach in all settings now it is realized that many parts of Africa are characterized by low malaria transmission intensity [7,8] and transmission intensity is decreasing in many areas in sub-Saharan Africa [9-12]. In such settings, presumptive treatment will lead to substantial over-treatment [8], which is particularly important now the relatively expensive artemisinin-combination therapy (ACT) is endorsed as first-line treatment [13-15]. Beside expenses, such practice will lead to a reduced susceptibility of parasites to ACT because of the uncontrolled use of artemisinins [16]. It is important that diagnosis is established before ACT is prescribed.

Microscopy is the gold standard method for diagnosis of malaria, but the provision of microscopic facilities may not always result in accurate diagnosis [2,8,14,17]. Malaria rapid diagnostic testing (RDT) forms an attractive alternative to routine microscopy with good sensitivity and specificity profiles [1,18] and more objective results. Wide-scale implementation of RDTs may, therefore, improve malaria diagnosis. This was acknowledged by the Tanzanian Ministry of Health and Social Welfare. The Kagera Region was the first in Tanzania where a policy change was implemented as a pilot to tackle the problem of malaria over-diagnosis. Malaria rapid diagnostic testing replaced routine microscopy as the main diagnostic tool and clinicians were advised to restrict treatment to RDT positive individuals only.

Here, the impact of the policy change was assessed by describing diagnostic behaviour and treatment decision-making in febrile outpatients under 10 years of age in three hospitals in the Kagera and Mwanza Region in Tanzania. Transmission intensity in these areas, all nearby Lake Victoria, was assessed by serological markers of malaria exposure.

## Methods

### Study site

In this study, common practices in fever diagnosis in the outpatient departments were described for three designated district hospitals (DDH) in northern Tanzania: Biharamulo DDH and Rubya DDH, both situated in the Kagera Region, and Sumve DDH, situated in the Mwanza Region. Biharamulo is situated at 1,490 m, Rubya at >1,500 m and Sumve at 1,100 m. The transmission intensity of the catchment areas is suspected to be different based on their distance to Lake Victoria and altitude [19]. However, no formal assessments of transmission intensity have been reported.

Healthcare at these outpatient departments was largely provided by clinical officers with two to three years' clinical training. Pneumonia, HIV, tuberculosis, gastrointestinal infections, various parasite infections and malaria are common conditions in outpatients according to hospital records. Malaria transmission is related to the long rains in March-April and the less reliable short rains in October -November. The study was approved by the Kilimanjaro Christian Medical Centre Research Ethics Committee. Written informed consent was obtained from parents or care-takers of all participants.

### Prospective data before and after policy change

Data were prospectively gathered from all patients under the age of 10 years presenting at the outpatient department of Sumve DDH with reported or measured fever in the months September 2009 - January 2010. In Biharamulo DDH and Rubya DDH, identical data were collected in September - October 2009. This is the period before the policy change when microscopy was the only available tool to diagnose malaria. At the end of October 2009, the Kagera Region was the first region in Tanzania where a policy change was implemented by the National Malaria Control Programme (NMCP) of the Ministry of Health and Social Welfare. This policy change comprised the replacement of microscopy as the main diagnostic tool for malaria by RDTs (ICT Malaria Pf Cassette Test (ML01), ICT Diagnostics) with a sensitivity of 82% at parasite densities of 200 parasites/μL, of 97% for parasite densities >2000 parasites/μL and a false positivity rate of ~1% [20]. Clinical officers were now strictly advised to only treat RDT positive individuals with anti-malarials; the advice was to look for alternative diagnoses in RDT-negative individuals. The medical officers in charge received training from government officials about the implementation of the policy change. After this, each medical officer in charge introduced the policy change during a two-day workshop to the clinical officers. To evaluate the short-term impact of this policy change, data were gathered for a second period in Biharamulo DDH (January 2010 - February 2010) and Rubya DDH (October 2009 - February 2010).

The same information was collected before and after policy change. The following information was collected in a questionnaire: age, gender, month attending the hospital, resident or no resident of the area in which the hospital was situated, bed net use, use of medication in the previous two weeks, auxiliary temperature, clinical diagnosis made by a clinical officer, request for blood slide, result of blood slide, treatment given in the hospital, and result of RDT. In Biharamulo and Rubya another question 'request for RDT' was added in the second period. Auxiliary temperature was measured with digital thermometers; blood slides were prepared according to hospital routine, stained with 10% Giemsa solution and read by a single experienced microscopist.

Before the policy change, a finger prick blood sample was taken for malaria parasite detection by RDT (Paracheck Pf Rapid test, Orchid Biomedical Systems) in each patient enrolled for an objective assessment of infection with *Plasmodium falciparum*. The RDT was not part of hospital routine, but was used to facilitate objective diagnosis for the current study. The RDT was used according to the manufacturer's instructions and is based on the detection of histidine-rich protein II. This RDT has a sensitivity of 75% at parasite densities of 200 parasites/μL, of 100% for parasite densities >2,000 parasites/μL and a false positivity rate of ~7% [20]. The results of the RDTs were available for clinical officers for diagnosis.

### Transmission intensity

For an objective assessment of exposure to malaria, serum samples were collected using a single finger prick (~20 μL) from all individuals, both adults and children, attending these three hospitals in Northern Tanzania in November 2009. This group included patients, people coming for routine follow-up visits and people accompanying patients. This sampling method has previously been validated to obtain a selection of samples representative of the general population in the hospital catchment areas [21]. Information was collected on age, gender, area of residency, bed net use, and use of medication in the previous two weeks.

### Assessment of malaria exposure by enzyme-linked immunosorbent assay (ELISA)

Serum was eluted from filter papers as described by Corran *et al *[22]. Immunoglobulin G antibodies against blood stage antigens were detected by indirect ELISA, as previously described in detail [22,23] using recombinant Apical Membrane Antigen-1 (AMA-1). AMA-1 was suggested as a suitable antigen for areas of moderate or low transmission intensity because of its high immunogenicity that results in saturating responses at high endemicity but a good discriminating power at low endemicity [24]. To generate an OD cut-off value above which samples were deemed antibody positive, the distribution of OD values was fitted as the sum of two Gaussian distributions (assuming a narrow distribution of seronegatives and a broader distribution of seropositives) using maximum likelihood methods [22].

### Data analysis

Statistical analyses of data were performed using SPSS version 18.0.0 and Stata 9.2 (Stata Corp, College Station TX, USA). Categorical variables were compared between groups by the Pearson Chi-square test or Fisher's Exact test, odds ratios (OR) with 95% confidence intervals (95% CI) were calculated. The main outcomes of the analyses were the proportion of individuals treated with anti-malarials despite a negative RDT and the proportion of individuals who did not receive anti-malarials despite a positive RDT. AMA-1 ELISA data were used to generate an age-seroprevalence plot. OD values were expressed as percentage of the positive control (normalized OD). The annual seroconversion rate (SCR), λ was estimated by fitting a simple model of the acquisition and loss of antibodies to the age-specific prevalence of the antibodies using maximum likelihood methods assuming a binomial distribution [23]. If visual examination of SCR suggested it was not uniform over the whole population (i.e. there was an obvious step in the age seroprevalence-data), models allowing for two forces of infection profile and profile likelihood plots were run to determine when the most likely time for change in transmission intensity occurred [21,25]. These resulted in a predicted time of change in transmission, which was incorporated in the catalytic model to generate estimates for previous and current SRC. Models allowing for two forces of infection were preferred if the fit compared to the single force model was significantly better by likelihood ratio test at a p < 0.05.

## Results

### Collected data

From September 2009 - February 2010, a total of 1,608 outpatients below 10 years of age presenting with (reported) fever were included. Age, reported bed net use and socio-demographic factors were similar between the three clinics (Table 1). The proportion of individuals with current fever was lowest in Biharamulo.

**Table 1:** Characteristics of the study population

	Sumve DDH	Biharamulo DDH		Rubya DDH	
		**Before policy change**	**After policy change**	**Before policy change**	**After policy change**

Study period	Sep '09 - Jan '10	Sep '09 - Oct '09	Jan '10 - Feb '10	Sep '09 - Oct '09	Oct '09 - Feb '10
Number of observations	362	360	135	250	501
Age					
Median (IQR)	15.0 (9.0 - 24.0)	15.0 (9.0 - 27.0)	14.5 (8.0 - 25.8)	12.0 (8.8 - 24.0)	14.0 (8.0 - 24.0)
Temperature					
Mean (sd)	37.5 (1.0)	37.0 (1.0)	37.4 (1.0)	37.8 (1.0)	37.7 (1.1)
Fever (≥37.8°C), % (n/N)	39.2 (135/344)	19.1 (68/356)	29.2 (38/130)	48.4 (121/250)	46.9 (235/501)
Reported use of bed net, % (n/N)	95.8 (340/355)	98.9 (356/360)	95.5 (128/134)	95.6 (239/250)	95.2 (477/501)
Reported anti-malarial use in preceding two weeks, % (n/N)	26.5 (95/358)	74.0 (361/488)		6.4 (48/751)	
Number of serum samples collected	308	300		320	

### Malaria case management before and after malaria treatment policy change

**Figure 1:**
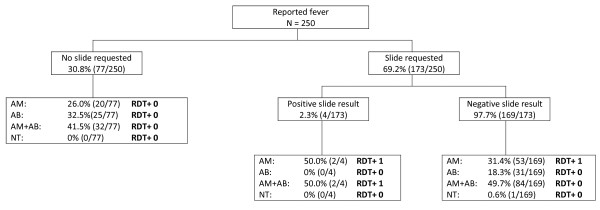
**Rubya DDH before policy change**. AM = anti-malarial treatment given; AB = antibiotics given; NT = no treatment installed; RDT = rapid diagnostic test.

**Figure 2:**
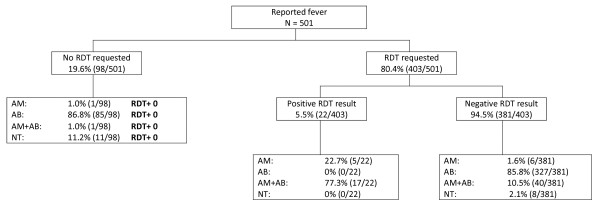
**Rubya DDH after policy change**. AM = anti-malarial treatment given; AB = antibiotics given; NT = no treatment installed; RDT = rapid diagnostic test.

Diagnostic behaviour and clinical management in Rubya DDH are summarized in Figures 1 and 2; the same information for Biharamulo DDH and Sumve DDH is shown in Additional file 1, Additional file 2 and Additional file 3. Treatment prescribing behaviour before and after the policy change in relation to parasite prevalence is shown in Table 2 for all three clinics. There was a striking difference in RDT parasite prevalence between the three clinics with the lowest parasite prevalence by RDT in Rubya (1-4%) and the highest in Sumve (42%, 152/362). Although the majority of RDT-positive individuals received anti-malarial treatment, in Biharamulo DDH, 5 malaria positive individuals did not receive anti-malarial treatment before policy change (8%, 5/67) compared to 2 (25%, 2/8) after policy change. In the other health centres, a positive RDT was followed by anti-malarial treatment in all instances. In all clinics a substantial proportion of slide negative patients received anti-malarial treatment before the policy change (Sumve 67%, Biharamulo 100%, Rubya 81.1%) and a substantial proportion of RDT negative patients received anti-malarial treatment (Sumve 68.1%, Biharamulo 89.1%, Rubya 76.9%). After the policy change this decreased to 38.7% in Biharamulo DDH and 10.0% in Rubya DDH.

**Table 2:** Treatment practices in relation to malaria parasite prevalence by rapid diagnostic test (RDT)

	Sumve DDH	Biharamulo DDH		Rubya DDH	
		**Before policy change**	**After policy change**	**Before policy change**	**After policy change**

Reported fever	362	360	135	250	501
Proportion RDT+, % (n/N)	42.0% (152/362)	19.6% (67/341)	6.3% (8/127)	1.2% (3/250)	4.4% (22/501)
Anti-malarial treatment					
RDT+, % (n/N)*	100% (152/152)	92% (62/67)	75.0% (6/8)	100% (3/3)	100% (22/22)
RDT-,% (n/N) **	68.1% (143/210)	89.1% (244/274)	38.7% (46/119)	76.9% (190/247)	10.0% (48/479)
Antibiotic treatment					
RDT+, % (n/N) ^#^	28.9% (44/152)	16.4% (11/67)	25.0% (2/8)	33.3% (1/3)	77.3% (17/22)
RDT-, % (n/N) ^##^	63.3% (133/210)	12.8% (35/274)	59.7% (71/119)	70.0% (173/247)	94.6% (453/479)

* proportion of RDT+ individuals who received anti-malarial treatment

** proportion of RDT- individuals who received anti-malarial treatment

^# ^proportion of RDT+ individuals who received antibiotics

^## ^proportion of RDT- individuals who received antibiotics

There was also a striking decrease in presumptive treatment of reported fever with anti-malarials. In Biharamulo DDH 89.0% (284/319) of patients without a slide taken was treated with anti-malarials before the policy change compared to 27.0% (27/100) of patients without a RDT after the policy change. In Rubya DDH this proportion changed from 67.5% (52/77) to 2.0% (2/98), indicating presumptive treatment was largely abandoned after the policy change.

### Management of non-malaria fevers before and after malaria treatment policy change

Together with the decrease in anti-malarial prescription, there was an increase in the proportion of individuals who received antibiotics. These were mostly broad-spectrum antibiotics like co-trimoxazole, ciproxin or amoxicillin. In RDT negative patients in Biharamulo DDH 12.8% received antibiotics before and 59.7% after the policy change. In Rubya DDH the number increased from 70.0% to 94.6%.

### The estimated transmission intensity generated from seroprevalence data

**Figure 3:**
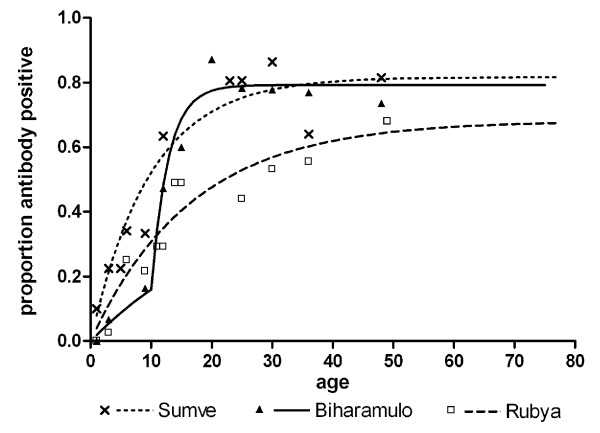
**Age specific seroprevalence plots for AMA-1 for Sumve, Biharamulo and Rubya**. For Biharamulo two forces of infection were fitted for the other two sites one force of infection fitted the data best. The seroconversion rate (SCR, λ) for Sumve was 0.082 (95% CI 0.063-0.11). For Biharamulo the SCR was 0.34 (95% CI 0.19-0.61) for the period up to 1999 (i.e. for individuals older than 10 years of age) but 0.019 (95% CI 0.011-0.035) for the period 1999-2009. The SCR for Rubya was 0.041 (95% CI 0.029 - 0.058).

From Biharamulo DDH, Rubya DDH, and Sumve DDH, a total of 298, 320 and 304 serum samples were successfully tested in the AMA-1 ELISA, respectively. The overall seroprevalence of AMA-1 antibodies was 52.0% (158/304), 40.3% (129/320), and 56.7% (169/298); there was a clear increase in seroprevalence with age in all sites (Figure 3). The seroconversion rate was highest in Sumve (λ = 0.082, 95% CI 0.063-0.11) and lowest in Rubya (λ = 0.041, 95% CI 0.029 - 0.058), in line with parasite prevalence rates by RDT. In Biharamulo, malaria transmission intensity appeared to have changed over time. While transmission intensity used to be intense (λ = 0.34, 95% CI 0.19-0.61), the current SCR was similar to that of Rubya (λ = 0.019, 95% CI 0.011-0.035), indicating low transmission intensity.

## Discussion

In three study areas in the vicinity of Lake Victoria, a considerable overuse of anti-malarials was seen prior to a new government policy to incorporate RDTs in the diagnostic process. There was no evident association between the actual level of transmission intensity and drug-prescribing behaviour across the sites: two thirds of the RDT negative individuals received anti-malarial drugs in the area of lowest endemicity. An official policy change, where RDTs were provided and healthcare providers were advised to adhere to RDT results in prescribing drugs was followed by lower anti-malarial prescription rates and higher antibiotic prescription rates in parasite negative children. This study indicates that an official policy change may reduce the overuse of anti-malarials, resulting in a more rational drug-prescribing behaviour.

In this study, treatment policies are described in children with reported fever visiting outpatient departments of three district hospitals in regions of differing malaria transmission intensity. Baseline diagnostic behaviour was similar between hospitals and over-treatment, defined as anti-malarial treatment of RDT-negative individuals, ranged from 68 - 90%, which is similar to previous studies [8,13,14]. Prior to policy change, these RDT results were provided to have an objective indicator of malaria infection for this study. The RDT was not requested by the responsible clinical officer who mostly based his or her diagnosis on clinical symptoms alone and microscopic results were often ignored in the final decision-making. Over-treatment was particularly common in Rubya and Biharamulo, areas where parasite prevalence by RDT and transmission intensity by serological markers of malaria exposure indicated low current levels of malaria transmission intensity. Like other recent studies [1,8,14], this study shows a discrepancy between the perceived and actual levels of transmission intensity. While there was no evidence for recent changes in transmission intensity in Rubya, transmission intensity appeared to have dropped in Biharamulo in the last decade. The reason for this change in transmission is unknown, but has been observed before in northern Tanzania [21].

Presumptive treatment of fevers with anti-malarials may have a beneficial prophylactic effect that could also result in a reduction in malaria transmission by reducing the human infectious reservoir [6]. However, in a setting like Rubya where transmission intensity is low, this beneficial effect is unlikely. The diagnosis of malaria and according treatment may simply be a 'convenient' clinical strategy avoiding the more complicated search for other causes of the presenting illness [26]. Treatment of all febrile episodes as malaria is likely to result in underdiagnosis of other fever-causing disorders such as childhood pneumonia [2]. In addition, there are financial implications. Over-treatment will often be with the highly effective, but expensive, artemether-lumefantrine (AL). In the current study, all malaria episodes were treated with AL. AL and other ACTs are 10-times more expensive than previously used drugs as sulphadoxine-pyrimethamine [27,28] making reliable diagnosis crucial for cost-effective use [29]. Importantly, there is concern for a reduced susceptibility of *P. falciparum *parasites for ACT [30] and the spread of parasites with reduced susceptibility to ACT may be enhanced by irrational drug use [31]. Reports on allelic selection after artemether-lumefantrine over-use [32] provide additional warnings against over-use of ACT.

Microscopic examination of a blood smear is the gold standard method for the diagnosis of malaria, but even if microscopy is used, there can be substantial over-diagnosis of malaria [17]. In Sumve DDH and Rubya DDH clinicians frequently requested blood slides, but also frequently ignored negative results. In these hospitals up to 81% of the slide-negative individuals were treated with anti-malarials. In Biharamulo DDH 79% of the children were treated with anti-malarials while no lab-confirmation was sought (i.e. no slide was requested). The Tanzanian Ministry of Health and Social Welfare implemented a policy change in the Kagera Region to tackle malaria over-diagnosis and over-treatment by replacing routine microscopy with RDTs as main diagnostic tool. Although RDTs may not be 100% sensitive in detecting (low-density) malaria infections [1], a prospective study found that restricting anti-malarials to RDT-positive individuals is a safe strategy in low endemic areas that does not lead to excess mortality due to false-negative results [33]. RDT results are objective and can also be confirmed by the person responsible for the clinical decision making who may not be confident with microscopy. The two different RDTs used in this study are both based on HRP-2 for parasite detection and have similar sensitivity; we do not expect marginal differences in RDT sensitivity to have influenced the treatment prescribing behaviour. The results of this study indicate an evident short-term impact on diagnostic behaviour and treatment decisions, unlike results from a previous study in Zambia [34]. In Rubya DDH, the proportion of individuals receiving anti-malarials despite negative RDTs was reduced seven-fold. This indicates that clinicians appeared willing to trust the RDT result in their decision-making, at least shortly after receiving clear instructions from the Tanzanian Ministry of Health. Presumptive treatment was not completely abandoned but decreased substantially. It is encouraging that antibiotic prescription increased in RDT-negative individuals, indicating that alternative diagnoses and treatment options are considered.

The current study was purely observational and no attempts were made to change the clinical decision making process during the study. The new government guidelines were explained by the medical officers in charge and information on the sensitivity of RDTs was provided. Although an effect of the present study cannot be ruled out completely, similar observational studies showed no positive effect on diagnostic and drug-prescribing behaviour [8,14]. Therefore, the impact of the current observational study on treatment practices will have been negligible compared to the new guidelines provided by the government. There are conditions for the policy change to work and keep working. First of all, the hospital staff must be well trained and confident regarding the new policy and new diagnostic test and clear guidelines should also be provided on treatment policies for RDT negative individuals. Secondly, the RDTs must be available at all times, as well as anti-malarial drugs and antibiotics. In Rubya DDH, RDTs provided by the Tanzanian government were out of stock in the beginning of February 2010. This is also seen in other countries [34]. Consequently, this brings the risk of hospital staff returning to their former diagnostic and drug-prescribing behaviour.

## Conclusion

This study shows promising data on decrease of anti-malarial drug-prescription. Larger prospective studies are needed to confirm the current findings, to determine the impact of the new policy on morbidity and mortality and to assess the long-term impact of the described policy change. The current findings on rational drug-prescribing behaviour in Tanzanian hospitals are promising.

## Competing interests

The authors declare that they have no competing interests.

## Authors' contributions

GJHB and ES were responsible for data collection and were involved in study design, data analysis and manuscript preparation. AN carried out the indirect ELISA. MK was involved in study design. TB and SS were responsible for study design, data interpretation and manuscript preparation. All authors read and approved the final version of the manuscript.

## Supplementary Material

Additional file 1 **Biharamulo DDH before policy change**. Click here for file

Additional file 2 **Biharamulo DDH after policy change**. Click here for file

Additional file 3 **Sumve DDH (no policy change)**. Click here for file
