# Supplementary material for: Malaria diagnostic testing and treatment practices in three different Plasmodium falciparum transmission settings in Tanzania: before and after a government policy change
Source: Malar J. 2011 Apr 2;10:76. doi: 10.1186/1475-2875-10-76 (PMC3080800; doi:10.1186/1475-2875-10-76)
Supplement: Additional file 1 — Biharamulo DDH before policy change. [file 1475-2875-10-76-S1.DOC]

**Additional file 1: Biharamulo DDH before policy change**

|  | |  | | | | |  | | | |  | | |  | | | Reported fever  N = 360 | | | | | | | | | | |  | | | | |  | | | | |  | | | |  | | |  | | |
| --- | --- | --- | --- | --- | --- | --- | --- | --- | --- | --- | --- | --- | --- | --- | --- | --- | --- | --- | --- | --- | --- | --- | --- | --- | --- | --- | --- | --- | --- | --- | --- | --- | --- | --- | --- | --- | --- | --- | --- | --- | --- | --- | --- | --- | --- | --- | --- |
|  | | | |  |  | | | |  | | | |  | |  |  | |  | | |  | |  | | |  | | | |  | | | | | | |  | | | |  | | |  | | | |
|  | | | |  |  |  | |  |  | | | |  | |  | | |  | | |  | | | | |  | | | |  | |  | |  | | |  | | | |  | | |  | | | |
|  | | No slide requested  88.6% (319/360) | | | | | | | | |  | | |  | | |  | | |  | | | | |  | | | Slide requested  11.4% (41/360) | | | | | | | | | |  | | | |  | | |  | | |
|  | | | |  |  |  | | |  | | | |  | |  | | |  | | |  | | |  | |  | | | |  | |  | | | | |  | | | |  | | |  | | | |
|  |  | | |  |  |  | | |  | | |  |  | |  | | |  |  | |  | | |  | |  | | |  | | | | | |  | | | | |  | | |  | | | |  |
| AM:  AB: | | | 85.9% (274/319)  10.7% (34/319) | | | | | | | **RDT+ 48**  **RDT+ 4** | | | |  | | |  |  | | Positive slide result  31.3% (10/32) | | | | | | | |  | | |  | |  | | | | | Negative slide result  68.7% (22/32) | | | | | | |  | | |
| AM+AB:  NT: | | | 3.1% (10/319)  0.3% (1/319) | | | | | | | **RDT+ 5**  **RDT+ 1** | | | |  |  |  |  |  |  |  | | | |  | | | |  |  |  |  |  |  |  |  |  |  |  | | | | |  | |  |  |  |
|  |  |  |  |  |  |  |  |  |  |  |  |  |  |  |  |  |  |  |  |  |  |  |  |  |  |  |  |  |  |  |  |  |  |  |  |  |  |  |  |  |  |  |  |  |  |  |  |
|  | | |  | | | | | | |  | | | |  |  |  |  | AM:  AB:  AM+AB:  NT: | | | | 75.0% (6/8)  0% (0/8)  25.0% (2/8)  0% (0/8) | | | | | **RDT+ 4**  **RDT+ 0**  **RDT+ 2**  **RDT+ 0** | | | |  |  |  | | | AM:  AB:  AM+AB:  NT: | | | 100.0% (14/14)  0% (0/14)  0% (0/14)  0% (0/14) | | | | | | | **RDT+ 3**  **RDT+ 0**  **RDT+ 0**  **RDT+ 0** | |
|  | | | | | | | | | | | | |  | | | | | | | | | | | | | | | | | | | | | | | | | | | | | | | | | | |

RDT=rapid diagnostic test

AM= antimalarial treatment given

AB= antibiotics given

NT=no treatment installed
