# Supplementary material for: Malaria diagnostic testing and treatment practices in three different Plasmodium falciparum transmission settings in Tanzania: before and after a government policy change
Source: Malar J. 2011 Apr 2;10:76. doi: 10.1186/1475-2875-10-76 (PMC3080800; doi:10.1186/1475-2875-10-76)
Supplement: Additional file 2 — Biharamulo DDH after policy change. [file 1475-2875-10-76-S2.DOC]

**Additional file 2: Biharamulo DDH after policy change**

|  | |  | | | | |  | | | | |  | | |  | | | Reported fever  N = 135 | | | | | | | | | | |  | | | | |  | | | | |  | | |  | | |  | |
| --- | --- | --- | --- | --- | --- | --- | --- | --- | --- | --- | --- | --- | --- | --- | --- | --- | --- | --- | --- | --- | --- | --- | --- | --- | --- | --- | --- | --- | --- | --- | --- | --- | --- | --- | --- | --- | --- | --- | --- | --- | --- | --- | --- | --- | --- | --- |
|  | | | |  |  | | | | |  | | | |  | |  |  | |  | | |  | |  | | |  | | | |  | | | | | | |  | | |  | | |  | | |
|  | | | |  |  |  | |  | |  | | | |  | |  | | |  | | |  | | | | |  | | | |  | |  | |  | | |  | | |  | | |  | | |
|  | | No RDT requested  80% (108/135) | | | | | | | | | |  | | |  | | |  | | |  | | | | |  | | | RDT requested  20% (27/135) | | | | | | | | | |  | | |  | | |  | |
|  | | | |  |  |  | | | |  | | | |  | |  | | |  | | |  | | |  | |  | | | |  | |  | | | | |  | | |  | | |  | | |
|  |  | | |  |  |  | | | |  | | |  |  | |  | | |  |  | |  | | |  | |  | | |  | | | | | |  | | | |  | | |  | | |  |
| AM:  AB: | | | 20.0% (20/100)  58.0% (58/100) | | | | | | | | **RDT+ 2**  **RDT+ 2** | | | |  | | |  |  | | Positive RDT result  14.8% (4/27) | | | | | | | |  | | |  | |  | | | | | Negative RDT result  85.2% (23/27) | | | | | |  | |
| AM+AB:  NT: | | | 7.0% (7/100)  15.0% (15/100) | | | | | | | | **RDT+ 0**  **RDT+ 0** | | | |  |  |  |  |  |  |  | | | |  | | | |  |  |  |  |  |  |  |  |  |  |  | | | |  | |  |  |
|  |  |  |  |  |  |  |  |  |  |  |  |  |  |  |  |  |  |  |  |  |  |  |  |  |  |  |  |  |  |  |  |  |  |  |  |  |  |  |  |  |  |  |  |  |  |  |
|  | | |  | | | | | |  | | | | | |  |  |  |  | AM:  AB:  AM+AB:  NT: | | | | | | 100.0% (4/4)  0% (0/4)  0% (0/4)  0% (0/4) | | | | | | |  |  |  | | | AM:  AB:  AM+AB:  NT: | | | | | | 65.2% (15/23)  8.7% (2/23)  26.1% (6/23)  0% (0/23) | | | |
|  | | | | | | | | | | | | | |  | | | | | | | | | | | | | | | | | | | | | | | | | | | | | | | | |

RDT=rapid diagnostic test

AM= antimalarial treatment given

AB= antibiotics given

NT=no treatment installed
