# Supplementary material for: Malaria diagnostic testing and treatment practices in three different Plasmodium falciparum transmission settings in Tanzania: before and after a government policy change
Source: Malar J. 2011 Apr 2;10:76. doi: 10.1186/1475-2875-10-76 (PMC3080800; doi:10.1186/1475-2875-10-76)
Supplement: Additional file 3 — Sumve DDH (no policy change). [file 1475-2875-10-76-S3.DOC]

**Additional file 3: Sumve DDH (no policy change)**

|  | |  | | | | |  | | | |  | | |  | | | Reported fever  N = 362 | | | | | | | | | | | |  | | | | |  | | | | | |  | | | |  | | |  | | |
| --- | --- | --- | --- | --- | --- | --- | --- | --- | --- | --- | --- | --- | --- | --- | --- | --- | --- | --- | --- | --- | --- | --- | --- | --- | --- | --- | --- | --- | --- | --- | --- | --- | --- | --- | --- | --- | --- | --- | --- | --- | --- | --- | --- | --- | --- | --- | --- | --- | --- |
|  | | | |  |  | | | | |  | | |  | |  |  | | |  | | | |  |  | | |  | | | |  | | | | | | | |  | | | |  | | |  | | | |
|  | | | |  |  |  | |  | |  | | |  | |  | | | |  | | | |  | | | |  | | | |  | |  | | |  | | |  | | | |  | | |  | | | |
|  | | No slide requested  0.3% (1/362) | | | | | | | | |  | | |  | | |  | | | |  | | | | |  | | | Slide requested  99.7% (361/362) | | | | | | | | | | |  | | | |  | | |  | | |
|  | | | |  |  |  | | | |  | | |  | |  | | |  | | | | |  | |  | |  | | | |  | |  | | | | | |  | | | |  | | |  | | | |
|  |  | | |  |  |  | | | |  | |  |  | |  | | |  | |  | | |  | |  | |  | | |  | | | | | | |  | | | | |  | | |  | | | |  |
| AM:  AB: | | | 0% (0/1)  0% (0/1) | | | | | | **RDT+ 0**  **RDT+ 0** | | | | |  | | |  |  | | | Positive slide result  44.6% (161/361) | | | | | | | |  | | |  | | |  | | | | | Negative slide result  55.4% (200/361) | | | | | | |  | | |
| AM+AB:  NT: | | | 100% (1/1)  0% (0/1) | | | | | | **RDT+ 0**  **RDT+ 0** | | | | |  |  |  |  |  |  |  |  | | | |  | | | |  |  |  |  |  |  |  |  |  |  |  |  | | | | |  | |  |  |  |
|  |  |  |  |  |  |  |  |  |  |  |  |  |  |  |  |  |  |  |  |  |  |  |  |  |  |  |  |  |  |  |  |  |  |  |  |  |  |  |  |  |  |  |  |  |  |  |  |  |  |
|  | | |  | | | | | |  | | | | |  |  |  |  | AM:  AB:  AM+AB:  NT: | | | | 70.2% (113/161)  0.6% (1/161)  29.2% (47/161)  0% (0/161) | | | | | | **RDT+ 107**  **RDT+ 0**  **RDT+ 44**  **RDT+ 0** | | | | |  | | | | | AM:  AB:  AM+AB:  NT: | | | 34.0% (68/200)  31.0% (62/200)  33.0% (66/200)  2% (4/200) | | | | | | | **RDT+ 1**  **RDT+ 0**  **RDT+ 0**  **RDT+ 0** | |
|  | | | | | | | | | | | | |  | | | | | | | | | | | | | | | | | | | | | | | | | | | | | | | | | | | | |

RDT=rapid diagnostic test

AM= antimalarial treatment given

AB= antibiotics given

NT=no treatment installed
